# Editorials

**Published:** 1894-06

**Authors:** 


### HOMŒOPATHIC PHYSICIAN,

A MONTHLY JOURNAL OF

HOMŒOPATHIC MATERIA MEDICA AND CLINICAL MEDICINE.

"If our school ever gives up the strict inductive method of Hahnemann, we are lost, and deserve only to be mentioned as a caricature in the history of medicine."—CONSTANTINE HERING.

Vol. XIV. JUNE, 1894. No. 6.

### EDITORIALS.

IDEALISM IN MEDICINE.—When an artist sets out to paint a picture he creates in his own mind an imaginary conception of the painting such as he wishes it to appear when finished.

This *ideal* in a fine mind is much greater than his actual achievement upon the canvas. The greater the mind, the greater will be his ideal, and the greater will be the resulting work for the beholder. But it will fall short, far short, of the ideal creation in his imagination.

When a man sets out to perform any task, to overcome any obstacle, to vanquish an enemy, or suppress any evil, he unconsciously forms in his mind an ideal of the value and importance of the object to be attained—either for himself or for others. This stimulates him to vigorous effort to achieve success and encourages him under difficulties.

If he embrace any form of religion, he speedily erects in his own mind an ideal of the Supreme Being whom he worships, of the creed, the liturgy and the principles of righteousness inculcated. The ideal, therefore, is an ideal of perfection.

If he adopt any scientific principle or theory he similarly, by contemplation, conceives an ideal of perfection for that theory or principle.

Thus the creation of the ideal in one's mind is a habit that is almost unconscious. This habit may degenerate into a vice as is characteristic of the human mind, when by continual contemplation his *ideal* becomes an *idol* before which he prostrates himself, metaphorically speaking, in worship.

The gigantic size of his idol obscures the light that enters the temple of his mind, and immersed in the deep shadows it casts, he perceives not its defects, and resents warmly any criticism of its proportions, any proof of its falsity.

Thus by this mental process a man becomes an enthusiastic devotee of the cause he espouses, and a courageous defender of it against all assault.

The enthusiastic homœopathist, devoted to his ideal of the homœopathic method of treatment, is content to endure every kind of persecution and to suffer the shafts of ridicule and contempt for the sake of the cause he loves.

His search for the suitable medicine to meet the conditions in the cases he treats, involves the finding of a remedy having the *tout ensemble* of symptoms which appear in the patient. This is the ideal simillimum of the Hahnemannian worker which, if he can find, and apply, will bring about the desired recuperation with a promptness that seems miraculous. With this ideal of a perfect simillimum before him, he is inspired to the most exhaustive toil and the most patient waiting and watching. Thus a priceless service is rendered to the patient which he rarely appreciates and of which he may not even be conscious.

An ideal conception of his healing art degenerated into the vice of idolatry is the view of the homœopathist held by the allopathist who may be indulgent enough to look upon his vagaries with any charity at all, instead of the threatening hostility of the majority.

But after all, one of the prime causes of the hostility of the allopathist to the homœopathist is this very thing of idealism. Instructed in a system of medicine that appeals to the senses and is consistent with what is called common sense, the *regular* practitioner unconsciously erects in his own mind an ideal of pathology and therapeutics which is his idol, and which makes of him an intolerant bigot ready to exterminate his new-school antagonist.

This idealism, then, is one of the obstacles to an acceptance of homœopathic doctrine.

In the April number it was pointed out that one of the obstacles to the progress of Homœopathy was the habit of generalization. Now we find another obstacle in this habit of idealism. Are there any more? We shall see.

---

BACON'S CIPHER STORY.—In the book notices for this month appears a remarkable review of a remarkable new book, entitled *Bacon's Cipher Story.* The review is by a learned Shakespearean scholar, and we make no apology for introducing this strictly literary production into a journal devoted to medical topics.

The book claims to be a revelation that Shakespeare's plays were written by Sir Francis Bacon, and that into their composition he wove a cipher which, if unraveled, would reveal a startling and hitherto unknown history of the reign of Queen Elizabeth. Dr. Orville W. Owen, of Detroit, claims to have discovered this cipher and unraveled the story, and in this book gives it to the world.

The Rev. Mr. Tullidge in this review disputes his claim.

---

REMOVAL.—The readers of THE HOMŒOPATHIC PHYSICIAN will please take notice that the office of the journal has been removed to 1231 Locust Street, Philadelphia.

